# Targeting CoV-2 spike RBD and ACE-2 interaction with flavonoids of Anatolian propolis by in silico and in vitro studies in terms of possible COVID-19 therapeutics

**DOI:** 10.3906/biy-2104-5

**Published:** 2021-08-30

**Authors:** Halil İbrahim GÜLER, Fulya AY ŞAL, Zehra CAN, Yakup KARA, Oktay YILDIZ, Ali Osman BELDÜZ, Sabriye ÇANAKÇI, Sevgi KOLAYLI

**Affiliations:** 1 Department of Molecular Biology and Genetics, Faculty of Science, Karadeniz Technical University, Trabzon Turkey; 2 Department of Biology, Faculty of Science, Karadeniz Technical University, Trabzon Turkey; 3 School of Applied Sciences, Bayburt University, Bayburt Turkey; 4 Department of Chemistry, Faculty of Science, Karadeniz Technical University, Trabzon Turkey; 5 Department of Biochemistry, Faculty of Pharmacy, Basic Pharmaceutical Sciences, Karadeniz Technical University, Trabzon Turkey

**Keywords:** Propolis, Covid-19, SARS-CoV-2, pinocembrin, molecular docking

## Abstract

Propolis is a multi-functional bee product rich in polyphenols. In this study, the inhibitory effect of Anatolian propolis against SARS-coronavirus-2 (SARS-CoV-2) was investigated in vitro and in silico. Raw and commercial propolis samples were used, and both samples were found to be rich in caffeic acid, p-coumaric acid, ferulic acid, t-cinnamic acid, hesperetin, chrysin, pinocembrin, and caffeic acid phenethyl ester (CAPE) at HPLC-UV analysis. Ethanolic propolis extracts (EPE) were used in the ELISA screening test against the spike S1 protein (SARS-CoV-2): ACE-2 interaction for in vitro study. The binding energy values of these polyphenols to the SARS-CoV-2 spike and ACE-2 protein were calculated separately with a molecular docking study using the AutoDock 4.2.6 program. In addition, the pharmacokinetics and drug-likeness properties of these eight polyphenols were calculated according to the SwissADME tool. The binding energy value of pinocembrin was highest in both receptors, followed by chrysin, CAPE, and hesperetin. Based on the in silico modeling and ADME (absorption, distribution, metabolism, and excretion) behaviors of the eight polyphenols, the compounds exhibited the potential ability to act effectively as novel drugs. The findings of both studies showed that propolis has a high inhibitory potential against the Covid-19 virus. However, further studies are now needed.

## 1. Introduction

Severe acute respiratory syndrome (SARS) coronavirus-2 (SARS-CoV-2) is responsible for the coronavirus (COVID-19) pandemic. Coronavirus ranks as the seventh largest family infecting humans after SARS coronavirus and Middle East respiratory syndrome (MERS) coronavirus (Bachevski et al., 2020; Zhu et al., 2020). Compared with other viruses, coronavirus has high transmissibility and infectivity. It is mostly spread through the respiratory tracts and is transmitted directly or indirectly, generally through the mucous membranes, nose, mouth and eyes. Until an effective vaccine or medicine was found, many physical and chemical solutions have been used for protection against this virus. Facemasks, social distancing, and hygiene are the most widely used physical protective agents. A number of natural food supplements and vitamins are also used for strengthening the immune system, particularly vitamins D and C, and propolis (Bachevski et al., 2020; Scorza et al., 2020). 

Propolis is a resinous honeybee product obtained from beehives as a raw material. Honeybees mostly collect propolis from the tree leaves, bark and trunk, then transform it with various secretions and store it in the hive. Honeybees benefit from propolis in physical, chemical and biological terms (Bankova et al., 2019). They particularly use it for antiseptic, antimicrobial, antiviral, antioxidant, and antitumoral purposes. Propolis has been also extensively employed in traditional and complementary medicine on account of these wide-ranging biological activities (Pasupuleti et al., 2017). Pharmacological and biochemical studies in the last 30 years have shown that propolis has a wide range of biologically active properties such as antibacterial, antiviral, anti-inflammatory, antitumoral, hepatoprotective, neuroprotective activities and immunity enhancement in apitherapeutic applications (Pasupuleti et al., 2017; Bankova et al., 2019; Kolayli et al., 2020). 

Although its composition and bioactive properties depend on the flora of the area where it is collected, propolis consists of approximately 50% resin and balsam, and 30% wax, while the rest is composed of essential oils and aromatic compounds (Aliyazıcıoglu et al., 2011; Bankova et al. 2019; Kızıltas and Erkan, 2020). The active ingredients of propolis, which contains approximately 300 different organic compounds, are various polyphenols and volatile compounds found in the balsamic part. Although propolis is partially extracted by dissolution in water, glycol, and vegetable oils, the optimal solvent is 60%–70% ethanol (Oroian et al., 2020). Many different commercial propolis extracts are currently available in different forms, such as drops, sprays, pills, pastilles, etc. Higher polyphenol or flavonoid-containing propolis samples are regarded as high quality (Oroion et al., 2020). Polyphenols are the largest class of phytochemical compounds, and polyphenol-rich diets have been associated with numerous health benefits. Studies strongly support the idea of the use of dietary polyphenols in the prevention of degenerative diseases, particularly cardiovascular and neurodegenerative diseases and cancer (Tsao, 2010; Pasupuleti et al., 2017). 

Propolis is an excellent natural antimicrobial and antiviral compound (Przybyłek and Karpiński, 2019). Many studies have shown that propolis exerts an antiviral effect against various DNA and RNA viruses, such as HIV,*Herpes simplex*, HSV-1, HSV-2, *para*-influenza virus, influenza virus type A and B, adenovirus, avian reovirus, Newcastle virus disease, bovine rotavirus, and pseudorabies virus (Bankova et al., 2014; Bachevskiet al., 2020). The first study investigating the antiviral activity of propolis against coronaviruses was conducted in 1990. One in vitro study investigated only the antiviral effects of five propolis flavonoids (chrysin, kaempferol, quercetin, acacetin and galangin), and quercetin was observed to exhibit dose-dependent antiviral activity (Debiaggi et al., 1990).

During the COVID-19 pandemic, propolis and other bee products have attracted renewed interest against SARS-CoV-2 infection, and various molecular docking studies have confirmed this. *In silico *studies have reported that some of the active ingredients of propolis, especially some flavonoids, have a higher binding potential than antiviral drugs (hydroxychloroquine and remdesivir) used in COVID-19 spike protein and ACE-2 (Mady et al., 2020; Shaldam et al., 2020; Güler and Kara, 2020; Guler et al., 2021). These studies have shown that the active components of propolis also exhibit high binding potential to cellular angiotensin-converting enzyme-2 (ACE-2) receptors and the serine protease TMPRSS2 and PAK1 signaling pathways (Beratta et al., 2020; Scorzaet al., 2020). A clinical study in which propolis tablets were administered to PCR-positive Covid-19 patients (400 and 800 mg) (3×1) for seven days together with placebo reported that propolis shortened hospitalization times (Silveira et al., 2021). Propolis also exhibits immunomodulatory and anti-thrombosis activities (Beratta et al., 2020), which are also crucial in combating the virus. In addition, propolis has been shown to inhibit the systemic inflammatory response and to protect hepatic and neuronal cells in acute septic shock (Korish and Arafa, 2011).

Although propolis is one of the most commonly used natural prophylactic agents during the pandemic, scientific studies on propolis are insufficient. The present study, therefore, investigated the inhibitory effect of Anatolian propolis against the COVID-19 virus for the first time in terms of the spike S1 protein (SARS-CoV-2): ACE-2 inhibitor screening ELISA test as an in vitro study.

## 2. Materials and methods

### 2.1. Chemicals

The COVID-19 spike protein: ACE-2 assay ELISA kit (Cat. No. 79954) was purchased from BPS Bioscience (San Diego, CA, USA), while gallic acid, protocatechuic acid p-OH benzoic acid, catechin, caffeic acid, syringic acid, epicatechin, p-coumaric acid, ferulic acid, rutin, myricetin, resveratrol, daidzein, luteolin, t-cinnamic acid, hesperetin, chrysin, pinocembrin, caffeic acid phenethyl ester (CAPE), FeSO_4_.7H_2_O, Folin–Ciocalteu’s phenol, diethyl ether, ethyl acetate, and acetonitrile were purchased from Sigma-Aldrich (Sigma-Aldrich Chemie, Munich, Germany). Daidzein was obtained from from Cayman Chemical (Michigan, USA) and ferric tripyridyltriazine (Fe-III-TPTZ), FeCI_3_, CH_3_CO_2_Na.3H_2_O, acetonitrile from Merck (Merck, Darmstadt, Germany).

### 2.2. Propolis samples

Two different propolis samples were used in this study. Both propolis samples are examples of Anatolian flora, one being prepared from raw Anatolian propolis, while the other was Anatolian propolis used commercially. Propolis samples from seven different regions (Van, Rize, Zonguldak, Muğla, Antalya, Diyarbakır, and Giresun) were mixed equally to obtain a homogeneous Anatolian propolis sample (P1). Briefly, to 3 g of the powdered raw propolis was added 30 mL 70% ethanol. This was then mixed in a shaker at a controlled speed for 24 h (Heidolp Promax 2020, Schwabach, Germany), and ultrasonic (Everest Ultrasonic, İstanbul, Turkey) extraction was applied for 30 min at a 99% power adjustment. The mixture was then filtered through 0.2 μm cellulose filters (Millipore, Bedford, MA, USA). The ethanolic propolis extract of the second sample selected from among commercial propolis samples (P2) and was supplied by Bee&You (Bee’O) (SBS Scientific Bio Solutions Inc., İstanbul, Turkey). The commercial propolis extract is sold in pharmacies and widely used for apitherapeutic purposes in Turkey. The solid amounts in both propolis extracts were calculated after evaporating the solvent and are expressed as mg / mL. And also, all analyses of the propolis samples were calculated as g extracts. 

### 2.3. Characterization of the propolis samples 

#### 2.3.1. Total phenolic compounds (TPC)

The total phenolic content (TPC) values of both samples were measured with Folin–Ciocalteu’s test using gallic acid (GA) as standard (Singleton et al., 1999). Briefly, 20 µL of six different propolis extracts, standard samples dilutions (from 0.500 mg/mL to 0.015 mg/ml), and 0.2 N 400 µL Folin reagents were mixed and completed to 5.0 mL with distilled water, and then vortexed. After 3 min incubation, 400 µL of Na_2_CO_3_(10%) was added and incubated at 25 °C. The absorbance was measured at 760 nm after 2-h incubation. The TPC was expressed in mg GAE/mL using a standard curve.

#### 2.3.2. Total flavonoid content 

Total flavonoid concentrations of the propolis samples were measured by the spectrophotometric method using quercetin as standard (Fukumoto and Mazza, 2000). Briefly, 250 mL of different propolis extracts and standard dilutions (from 0.500 mg/mL to 0.015 mg/mL), 50 mL of 10% Al(NO_3_)_3_, and 50mL of 1 M NH_4_.CH_3_COO were mixed and completed 3.0 mL with methanol (99%), vortexed and incubated at 25 °C for 40 min. After incubation, the absorbance was then measured against a blank at 415 nm. The total flavonoid concentration was expressed in mg QUE/g extract by the curve.

#### 2.3.3. Determination ferric reducing/antioxidant power (FRAP) 

The total antioxidant capacities of the samples were determined using the ferric reducing/antioxidant power assay (FRAP) (Benzie &Strain, 1999). First, working FRAP reagent (ferric tripyridyltriazine (Fe-III-TPTZ) was prepared fresh by mixing 300 mM (pH: 3.6) acetate buffer, 10 mM TPTZ, and 20 mM FeCl_3_ solutions in a ratio of 10: 1: 1. Before the samples test, a standard curve was prepared with 1000 µM stock FeSO_4_.7H_2_O solution by serial dilutions. Next, 1.500 mL of the FRAP reagent, 50 µL of sample and 50 µL methanol were mixed and incubated for 4 min at 37 °C, and the absorbance was read at 595 nm against a reagent blank containing distilled water. FRAP values were expressed in µmol FeSO_4_.7H_2_O equivalents/g extract.

#### 2.3.4. Determination of phenolic compositions by HPLC-UV

For preparation of the propolis extracts for chromatographic analysis, 10 mL of ethanolic extract was evaporated, and the residue was dissolved using 10 mL of pH2 purified water. The aqueous solution was extracted three times with 5 mL of diethyl ether (15 min, 200 rpm, 25 °C) and three times with ethyl acetate (15 min, 200 rpm, 25 °C). The organic phase, which was collected in a flask after each extraction, was evaporated. The residue was then dissolved in 2 mL of methanol, filtered through 0.45 µm filters, and given to the HPLC device for analysis. The phenolic content analysis of the samples was performed in triplicate.

Phenolic content analysis of the samples was performed at a 280 nm wavelength in the RP-HPLC system (EliteLaChrome; Hitachi, Tokyo, Japan) with a C18 column (150 mm * 4.6 mm, 5 μm; Fortis). In the analysis using 70% acetonitrile/water (A) and 2% acetic acid/water (B) as mobile phase, the injection volume was 20 µL, the flow rate was 1.00 mL/min, and the column temperature was 30 °C. The analysis was performed using a gradient program. The R2 values of the calibration curves of the 19 standard phenolic compounds used in the analysis ranged between 0.998 and 1.000. The phenolic compound concentrations were calculated in mg/100 g extracts.

### 2.4. Inhibition assay for Covid-19

The spike S1 (SARS-CoV-2): ACE-2 inhibitor screening colorimetric assay kit (Cat. No. 79954) was purchased from BPS Bioscience (79954, San Diego, CA USA). The colorimetric test is designed for screening and profiling inhibitors targeting the interaction between the spike protein of SARS-CoV-2 and ACE-2. The aim of the test is to determine the possible inhibitory potential of tested samples for ACE-2 receptor and spike protein S1 interaction. Using the kit protocol, the absorbance was read at 450 nm on a UV/Vis spectrophotometer microplate reader. The propolis and standard phenolic samples were diluted with 70% ethanol, and the Covid-19/ELISA test procedure was then applied. All tests were performed in triplicate. Inhibition values (IC_50_) of the propolis extracts were calculated as µg of the extracts, but the pure phenolic standards were calculated as mM.

### 2.5. Molecular docking studies

AutoDock 4.2.6 software for molecular docking studies was used to investigate the possible interactions of eight ligands and reference molecules with the target proteins. The crystal structures of ACE-2 (PDB ID: 6M0J-chain A, Res: 2.45 Å) and SARS-CoV-2 spike RBD (PDB ID: 6YLA-chain A, Res: 2.42 Å) were retrieved from RCSB protein data bank at http://www.rscb.org. In order to evaluate the prediction of accuracy of the binding affinity between ligands and two target proteins, the binding free energies (ΔG) were calculated for the crystal structures and the docking mode. The 3-D structures of all ligands (pinocembrin, chrysin, cape, hesperetin, ferulic acid, t-cinnamic acid, p-coumaric acid, and caffeic acid) and the reference molecule (hydroxychloroquine) were retrieved from the PubChem database (https://pubchem.ncbi.nlm.nih.gov/) in sdf format and then converted to pdb format using BIOVIA DS Visualizer software (Dassault Systèmes BIOVIA, 2016).The prepared ligands and proteins were used as input files for the AutoDock 4.2.6 software (Morris et al., 2009). A Lamarckian genetic algorithm method, implemented in the program AutoDock 4.2.6, was employed. After energy minimization, the water molecules were deleted, and the standard docking procedure was used for a rigid protein and a flexible ligand with torsion angles of 100 independent runs per ligand. The receptor grid was generated using the grid box panel in Autodock, by including active site amino acid residues (Tyr449, Asn487, Gly496, Thr500, Gly502 andTyr505) of the spike RBD (Lan et al., 2020). However, all docking experiments were performed as blind docking (referring to the use of a grid box, which is large enough to encompass any possible ligand-receptor complex) to determine any interaction between targets and ligands. A grid of 126, 126, and 126 points in x, y, and z directions was built with a grid spacing of 0.375 Å. The default settings of the software were applied for all other parameters. In order to predict the binding strength of all ligands, the ligand-protein docked complexes were analyzed based on minimum binding energy values and ligand interaction (hydrogen/hydrophobic) patterns. The final visualization of the docked structures was performed using BIOVIA Discovery Studio Visualizer 2018 (Dassault Systèmes BIOVIA, 2016).

### 2.6. Pharmacokinetics and drug-likeness properties (ADME prediction)

In order for a drug to be effective, it must reach its target in the body in sufficient concentration and remain in bioactive form long enough for the expected biological events to occur there. Drug development involves absorption, distribution, metabolism and excretion (ADME) at an increasingly earlier stage in the discovery process, at a stage when the compounds are abundant but access to physical samples is limited (Daina et al., 2017). The pharmacokinetics, drug-likeness, and medicinal chemistry properties of eight ligands were predicted using the SwissADME server. Important parameters related to ADME properties, such as Lipinski’s five rules, drug solubility, pharmacokinetic properties, molar refraction, and drug-likeliness were analyzed. The SMILES format retrieved from the PubChem Database of the relevant ligands was used as input for the analysis tool (Daina et al., 2017).

### 2.7 Statistical analyses

Statistical evaluations were carried out on SPSS version 11.5 software (IBM SPSS Statistics, Armonk, NY, USA). Descriptive statistics were expressed as mean ± standard deviation (SD). The correlation and difference analyses were performed with Duncan’s multiple range test. Significance was determined at p < 0.05.

## 3. Results 

### 3.1. Propolis analyses

Table 1 shows the analysis of the two Anatolian propolis samples, one prepared from raw Anatolian propolis (P1) and the other a commercially available product (P2). The solid matters of the ethanolic propolis extracts differed. The commercial sample (P2) contained nearly twice as much solid material as the other sample (P1). The pH values of both propolis samples were between 4.50 and 4.80, and both were acidic. TPC was 123.04 mg GAE/g extract in sample P1 and 203.10 mg GAE/g extract in sample P2. The phenolic compound content of the commercial sample (P2) was approximately two-fold higher than that in P1. Similarly, total flavonoid content also differed between the two samples, being measured at 62.03 mg QUE/g extract in P2 and 10.80 mg QUE/g extract in P1. The total antioxidant capacities of the samples were investigated only through FRAP assay, and the total antioxidant capacities were found similar. The phenolic profile results for the two ethanolic propolis samples are summarized in Table 2. As a result of the phenolic composition analyzes performed by HPLC-UV, it was found that both propolis samples contained similar types of phenolic compounds, but their concentrations were different. According to the phenolic standards examined in the present study, hesperetin was found in higher amounts in the commercial sample (P2), while pinosembrin, CAPE, and chrysin were found to be higher in the P1 sample.

**Table 1:** Analysis of two Anatolian propolis samples.

		Solid matter in the extract (mg/mL)	pH	Total phenolic content (mgGAE/g)	Total flavanoid content (mgQUE/g)	Total antioxidant capacity (FRAP)(µmolFeSO4/g)
P1	Ethanolic propolis extract	0.10 ± 0.00	4.80 ± 0.01	123.04 ± 0.20a	10.80 ± 0.30a	1414.00 ± 17.00a
P2	Commercial Propolis (BEE’O)©	0.20 ± 0.01	4.50 ± 0.01	203.40 ± 15.00b	62.03 ± 4.90b	1427.00 ± 27.00b

a, b letter(s) are significantly different (p < 0.05) by Duncan’s multiple range test.

**Table 2:** Phenolic profile of the EPE samples by HPLC-UV.

Phenolic Standards (mg/g)	(P1)	(P2)
Gallic acid	-	-
Protocathequic acid	-	-
p-OH Benzoic acid	-	-
Catechin	-	-
Caffeic acid	70.77	24.86
Syringic acid	-	-
Epicatechin	-	-
p-Coumaric acid	88.19	13.451
Ferulic Acid	37.85	7.29
Rutin	-	-
Myricetin	-	-
Resveratrol	-	
Daidzein	-	-
Luteolin	-	-
t-Cinnamic acid	51.05	2.60
Hesperetin	71.10	151.47
Chrysin	66.51	59.54
Pinocembrin	168.55	90.21
CAPE	326.87	158.41

(-): not detected, P1:Ethanolic propolis extract prepared by us, P2: Commercial propolis supplied by (BEE’O).

### 3.2. Molecular docking studies

**Figure 1:**
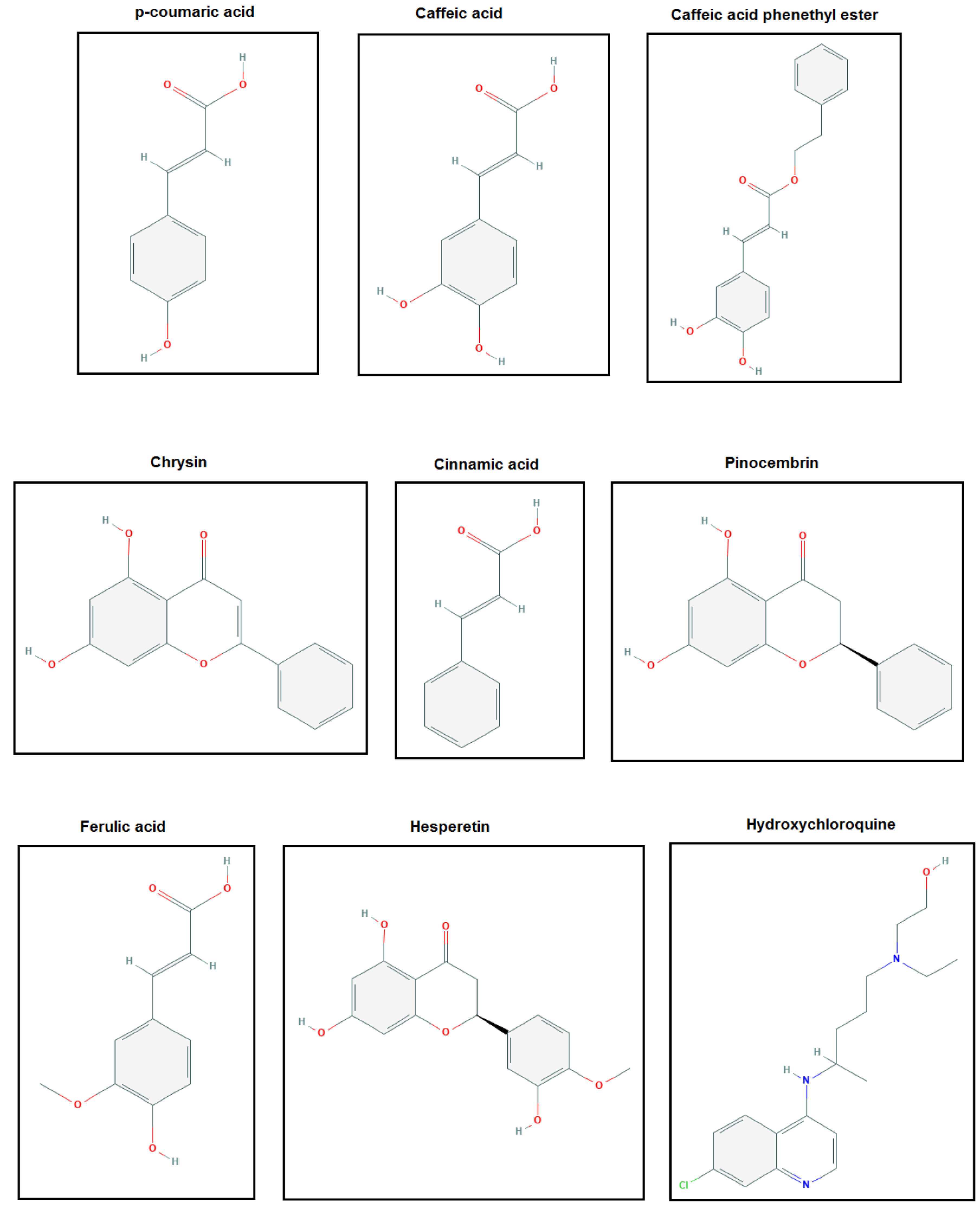
2-D structures of ligands used in the present study.

**Figure 2:**
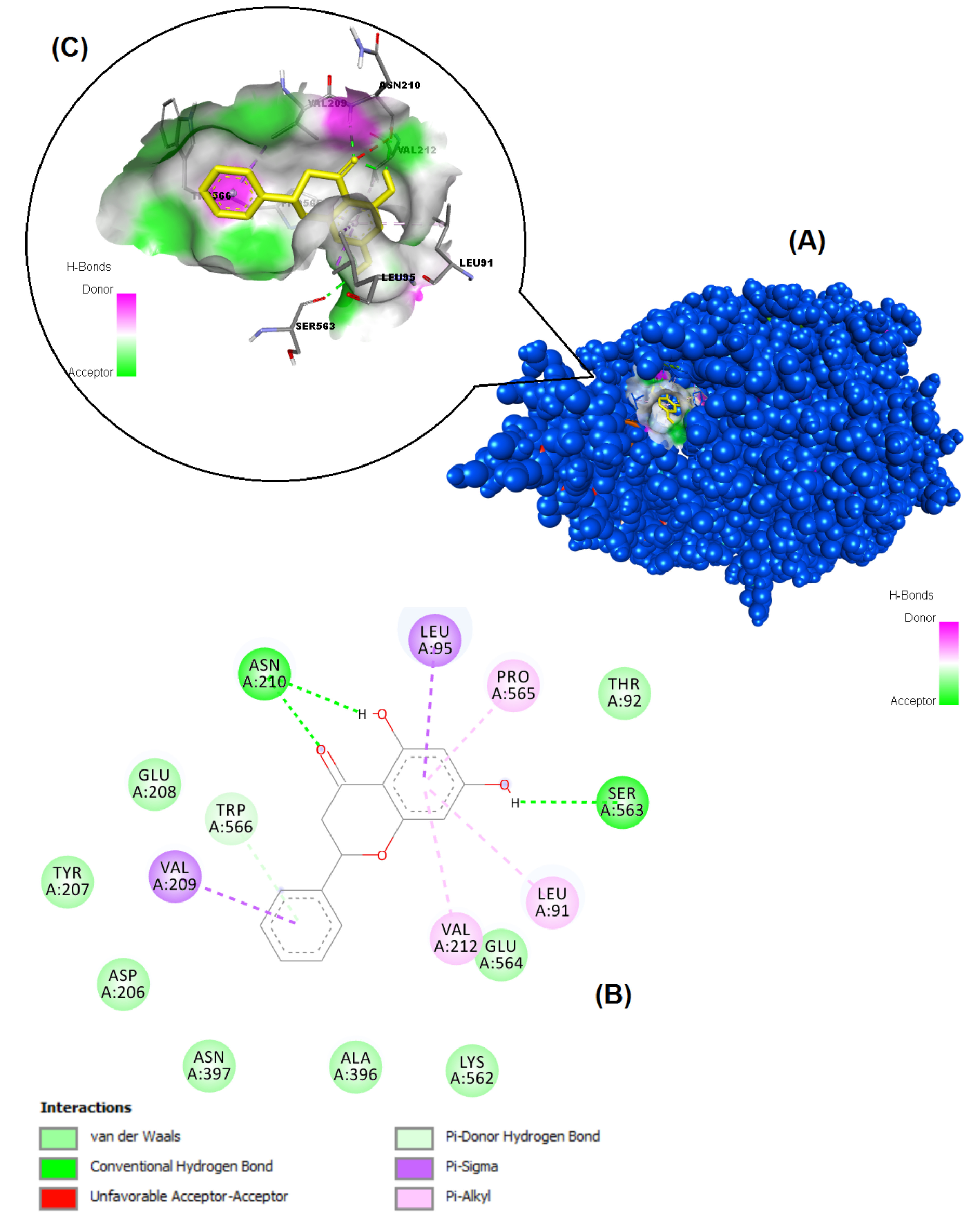
Binding pose profile of pinocembrin in the target protein ACE-2 (A), blue shaped molecule represents the receptor and yellow shaped molecule indicates the ligand. The two-dimension (2D) (B) and three-dimension (3D) (C) interactions analysis of ACE-2 protein with compound pinocembrin.

**Figure 3:**
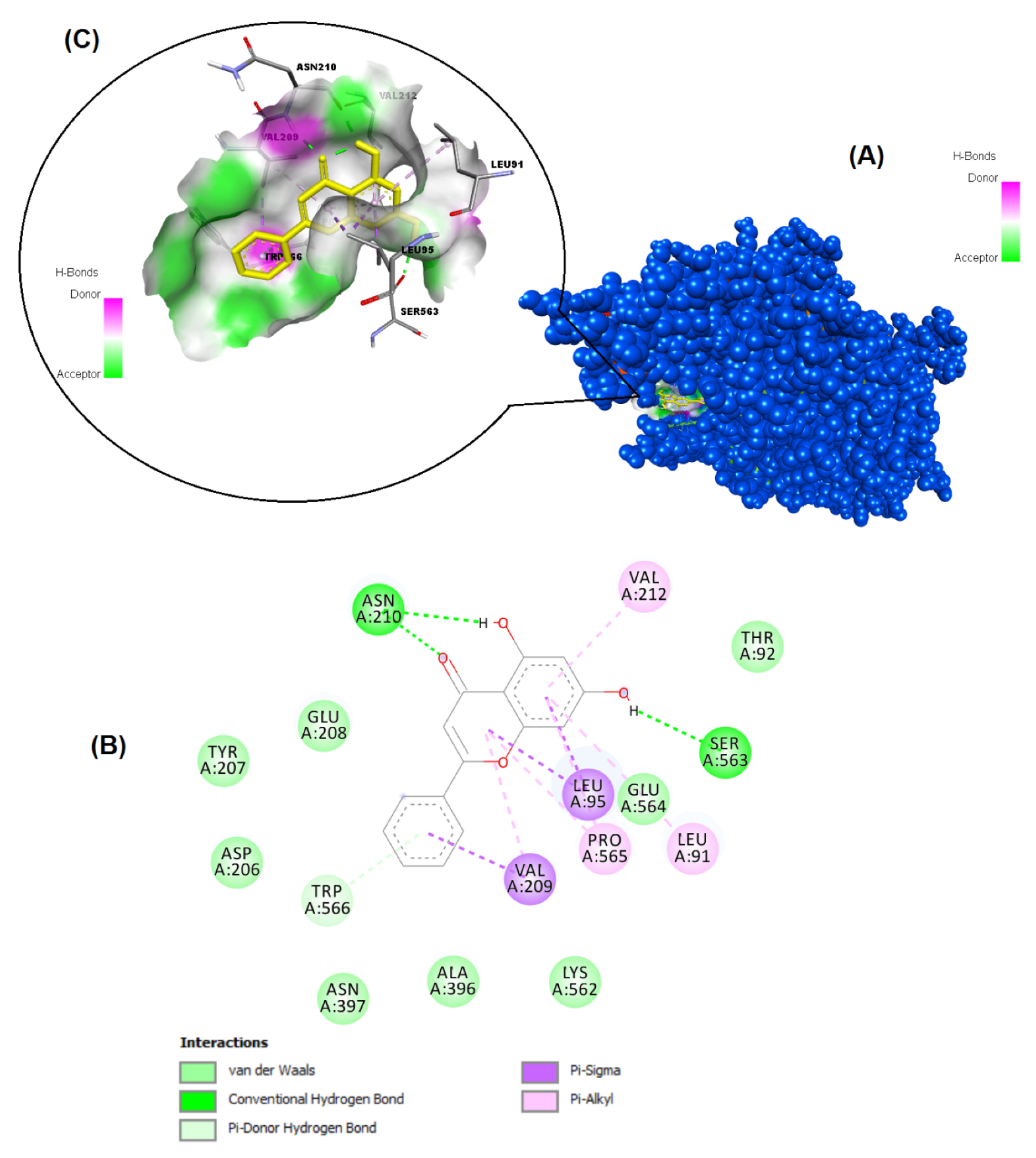
Binding pose profile of chrysin in the target protein ACE-2 (A), blue shaped molecule represents the receptor and yellow shaped molecule indicates the ligand. The two-dimension (2D) (B) and three-dimension (3D) (C) interactions analysis of ACE-2 protein with compound chrysin.

**Figure 4:**
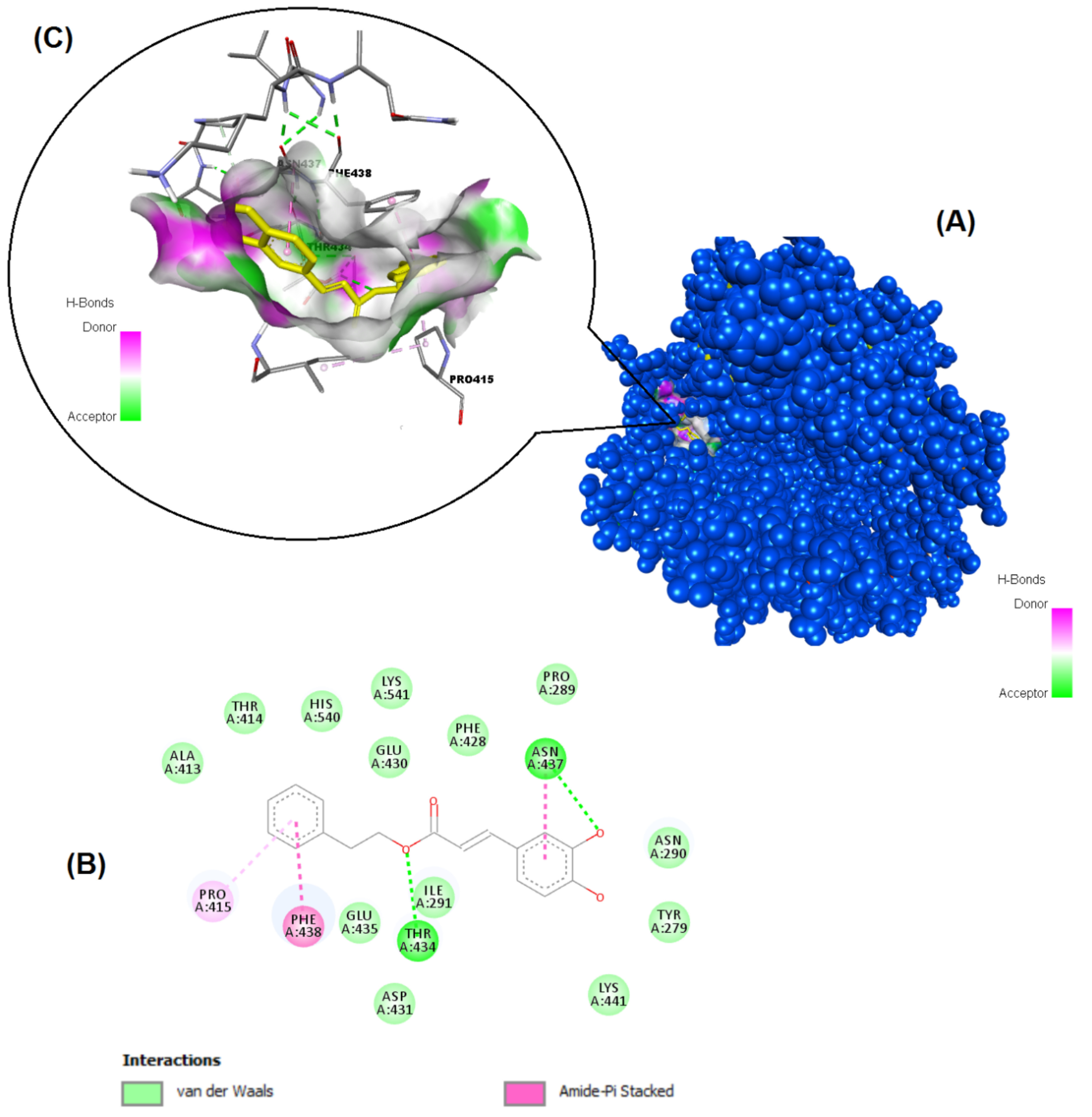
Binding pose profile of CAPE in the target protein ACE-2 (A), blue shaped molecule represents the receptor and yellow shaped molecule indicates the ligand. The two-dimension (2D) (B) and three-dimension (3D) (C) interactions analysis of ACE-2 protein with compound CAPE.

**Figure 5:**
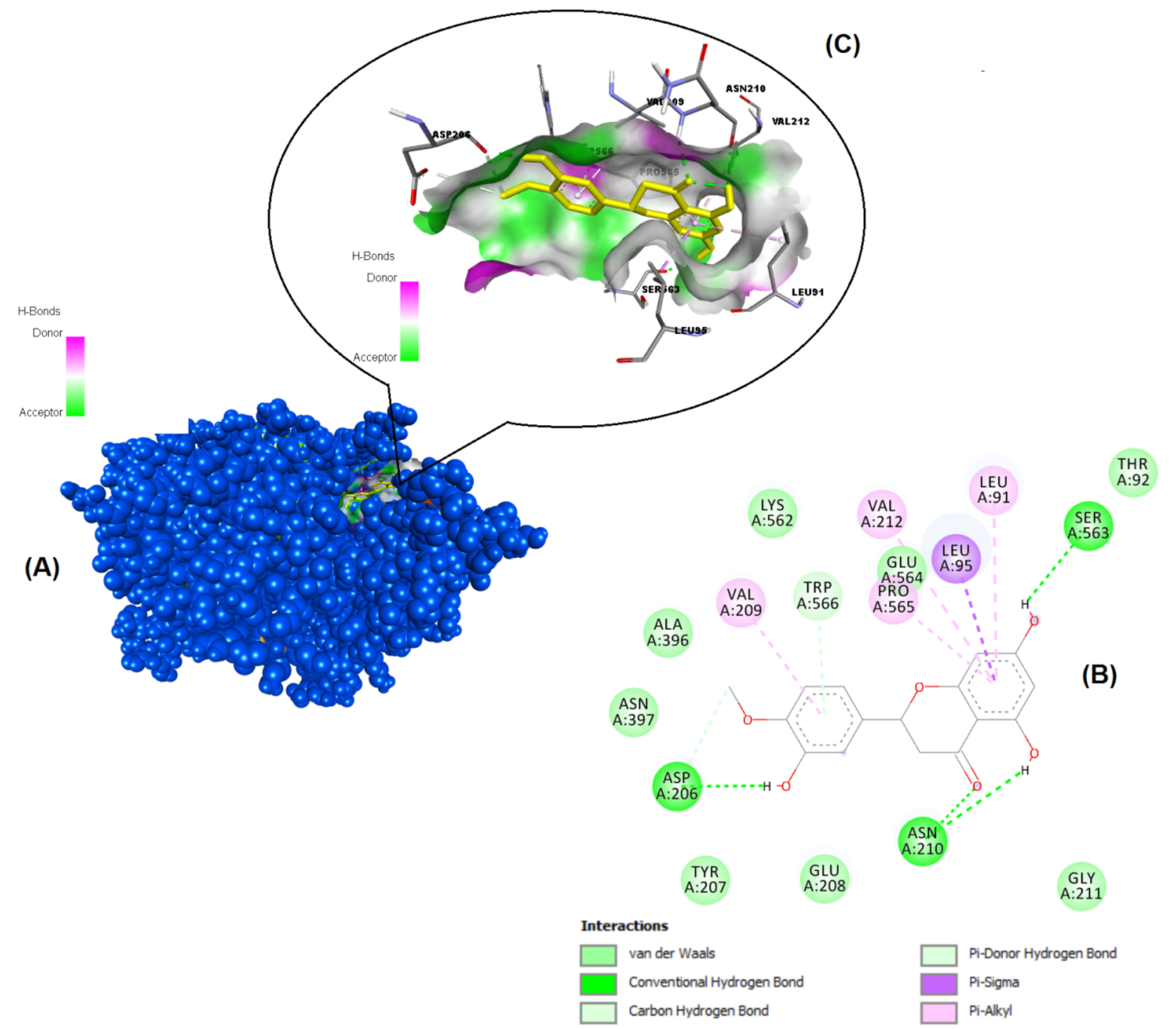
Binding pose profile of hesperetin in the target protein ACE-2 (A), blue shaped molecule represents the receptor and yellow shaped molecule indicates the ligand. The two-dimension (2D) (B) and three-dimension (3D) (C) interactions analysis of ACE-2 protein with compound hesperetin.

**Figure 6:**
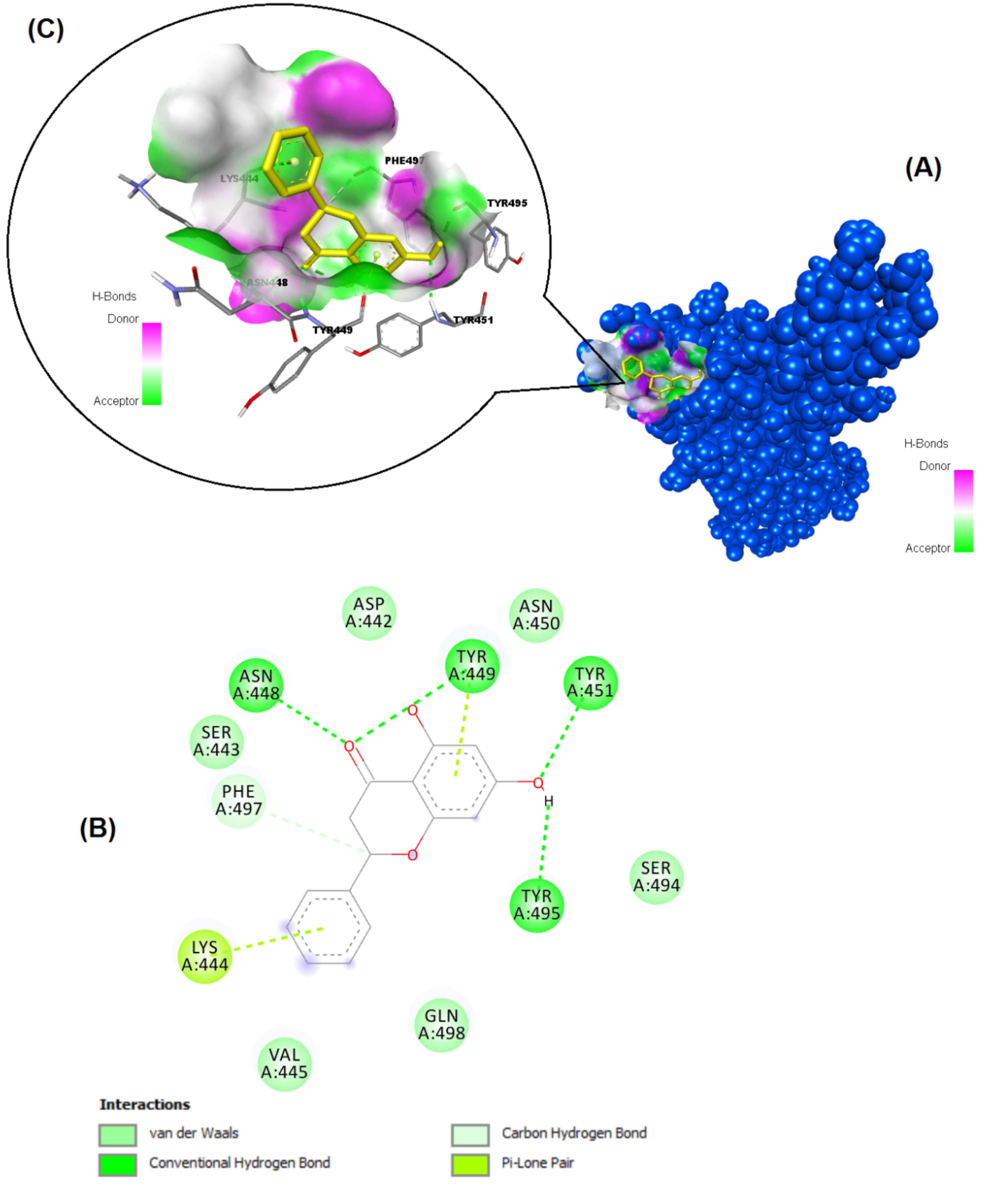
Binding pose profile of pinocembrin in the SARS-CoV-2 Spike receptor binding domain (A), blue shaped molecule represents the receptor and yellow shaped molecule indicates the ligand. The two-dimension (2D) (B) and threedimension (3D) (C) interactions analysis of SARS-CoV-2 Spike RBD with compound pinocembrin.

**Figure 7:**
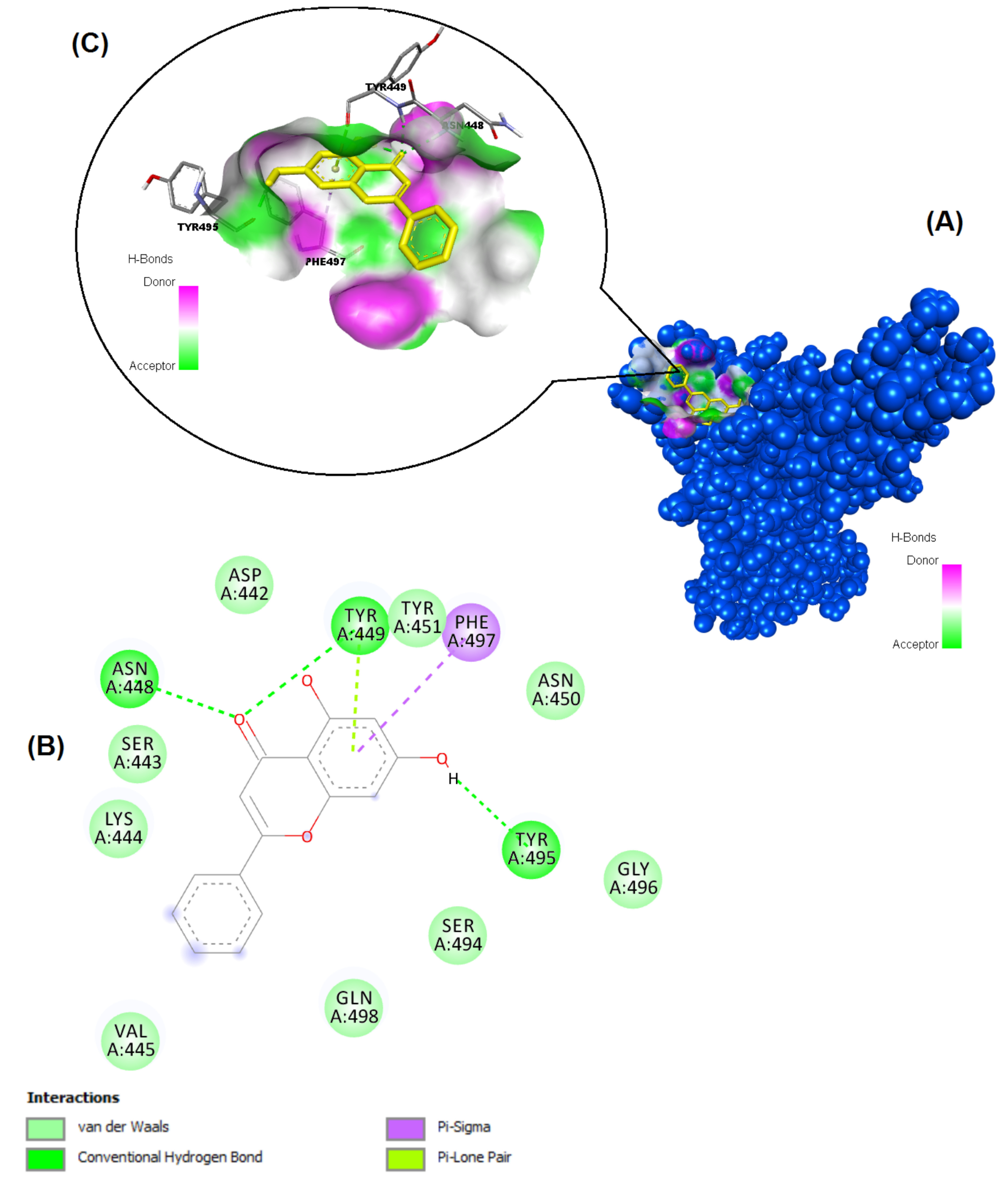
Binding pose profile of chrysin in the SARS-CoV-2 Spike receptor binding domain (A), blue shaped molecule represents the receptor and yellow shaped molecule indicates the ligand. The two-dimension (2D) (B) and three-dimension (3D) (C) interactions analysis of SARS-CoV-2 Spike RBD with compound chrysin.

**Figure 8:**
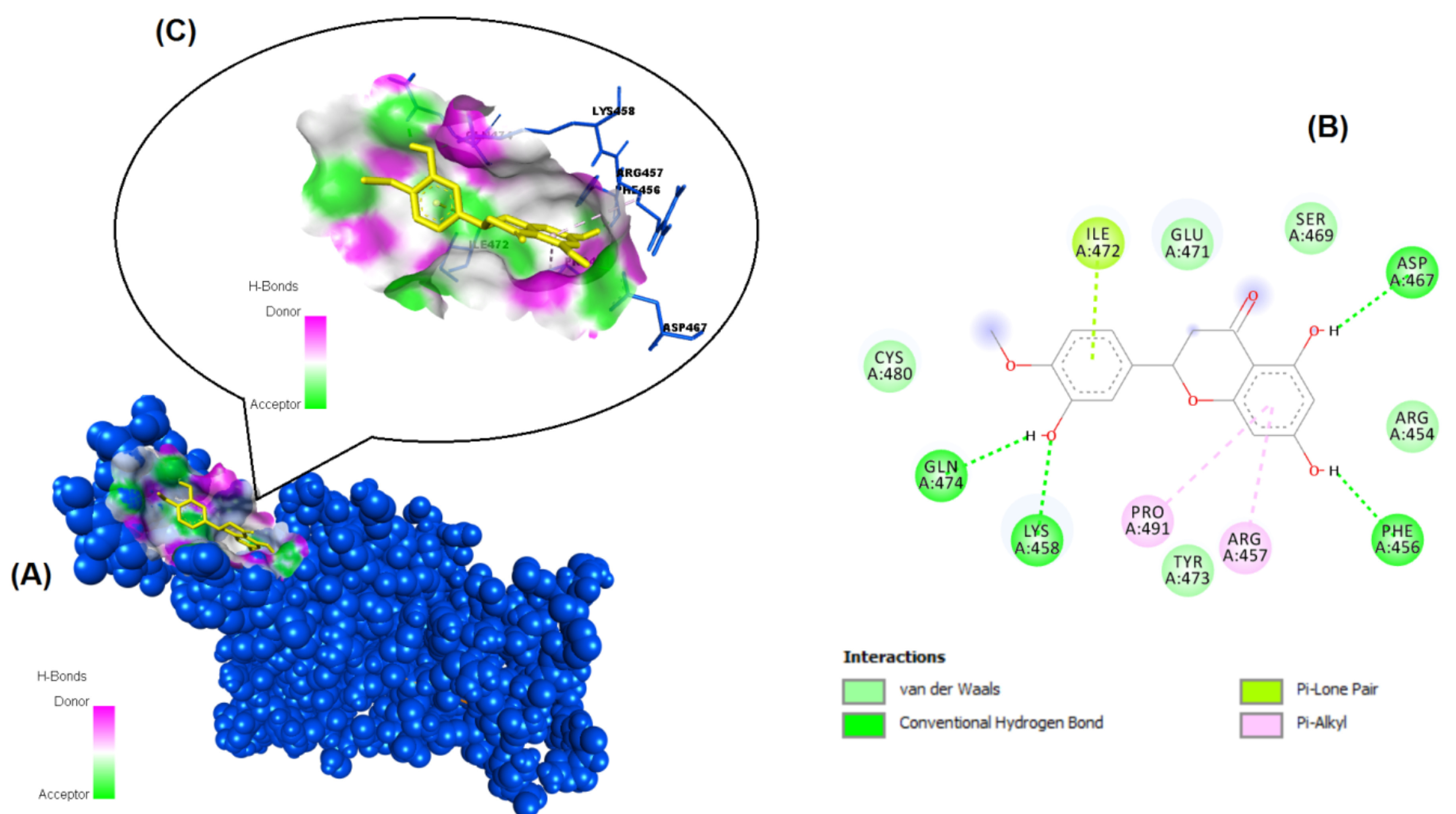
Binding pose profile of hesperetin in the SARS-CoV-2 Spike receptor binding domain (A), blue shaped molecule represents the receptor and yellow shaped molecule indicates the ligand. The two-dimension (2D) (B) and three-dimension (3D) (C) interactions analysis of SARS-CoV-2 Spike RBD with compound hesperetin.

**Figure 9:**
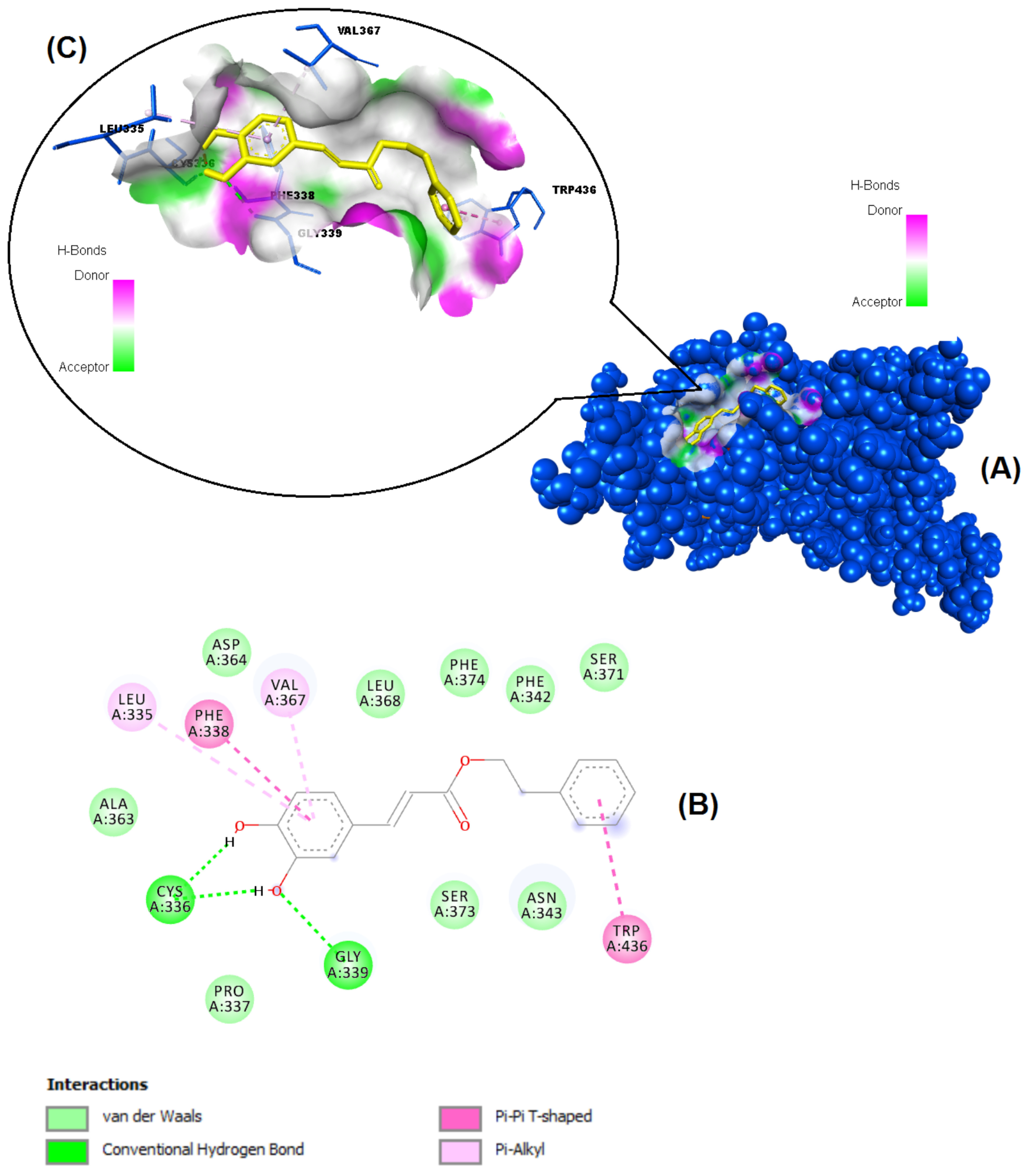
Binding pose profile of CAPE in the SARS-CoV-2 Spike receptor binding domain (A), blue shaped molecule represents the receptor and yellow shaped molecule indicates the ligand. The two-dimension (2D) (B) and three-dimension (3D) (C) interactions analysis of SARS-CoV-2 Spike RBD with compound CAPE.

The structures of the polyphenols used in the molecular docking program are given in Figure 1. The binding free energy values for ACE-2 and SARS-CoV-2 spike RBD were calculated using the AutoDock 4.2.6 program and are summarized in Table 3. The docked poses, interacting residues and interactions of the four ligands with the lowest binding energy with ACE-2 and SARS-CoV-2 spike RBD are given in Figures 2–9. Details concerning the estimated binding affinities (kcal/mol) and K _i_ values of docked ligands are shown in the Table 3. The results showed that four compounds (pinocembrin, chrysin, caffeic acid phenethyl ester, and hesperetin) had very low binding free energies to the ACE-2 receptor and SARS-CoV-2 spike Protein RBD. It was also observed that these four flavonoids have higher binding potentials than hydroxychloroquine, which was used as a Covid-19 drug and as the standard ligand in the present study. From the Table 3, it can be clearly predicted that pinocembrin has the highest binding energy value at –8.58 kcal/mol for ACE-2 protein and –7.54 kcal/mol for SARS-CoV-2 spike RBD, followed by chrysin, with dock scores of –8.47 and –7.48 kcal/mol, respectively. The interactions in Figures 6 and 7 show that pinocembrin and chrysin form two hydrogen bonds with Tyr449 residue in the active site of SARS-CoV-2 spike protein RBD.

**Table 3:** Summary of estimated binding affinity (kcal/mol) and Ki values of docked ligands against ACE-2 and SARS-CoV-2 spike receptor binding domain, and interacted residues in the binding sites.

Receptor name / PDB ID	Ligand name	Binding energy (kcal/mol)	Ki	Interacted residues with ligand
Angiotensin-Converting Enzyme-2 (ACE-2)EC: 3.4.17.23 /6M0J (Chain A)Res: 2.45 Å	Pinocembrin	–8.58	510.99 nM	Asn210, Leu9, Pro565, Ser563, Leu91, Val212, Val209
	Chrysin	–8.47	623.53 nM	Asn210, Val212, Ser563, Glu564, Leu91, Leu95, Pro565, Val209
	CAPE (Caffeic acid phenethyl ester)	–8.42	677.67 nM	Asn437, Ile291, Thr434, Phe438, Pro415
	Hesperetin	–8.22	943.94 nM	Leu91, Ser63, Asn210, Asp206, Val209, Trp566, Val212, Glu564, Pro565, Leu95
	Ferulic acid	–5.65	72.03 µM	His540, Ile291, Pro289, Thr434, Glu430
	t-Cinnamic acid	–5.65	72.06 µM	Leu456, Trp477, Leu503, Trp165, Trp271, Lys481
	p-coumaric acid	–5.63	74.21 µM	Trp165, Pro500, Leu503, Leu456, Trp477, Lys481, Trp271
	Caffeic acid	–5.31	127.93 µM	Leu73, Ala99, Leu100, Lys74, Asn103
	*Hydroxychloroquine	–7.90	1.61 µM	Arg393, Phe390, Leu391, Asn394, His378, His401, Asp350
SARS-CoV-2 spike receptor binding domain/6YLA (Chain A)Res: 2.42 Å	Pinocembrin	–7.54	2.99 µM	Asn448, Tyr449, Tyr451, Tyr495, Lys444, Phe497
	Chrysin	–7.48	3.29 µM	Asn448, Tyr449, Phe497, Tyr495
	Hesperetin	–7.28	4.63 µM	Ile472, Asp467, Phe456, Arg457, Pro491, Lys458, Gln474
	CAPE	–7.17	5.54 µM	Leu335, Phe338, Val367, Trp436, Gly339, Cys336
	Ferulic acid	–6.93	8.29 µM	Leu441, Tyr495
	t-Cinnamic acid	–6.64	13.57 µM	Phe497, Lys444, Asn448, Tyr449, Tyr495
	Caffeic acid	–6.43	19.36 µM	Leu441, Tyr495, Phe497
	p-coumaric acid	–5.97	42.06 µM	Phe497, Tyr495, Leu441
	*Hydroxychloroquine	–6.32	23.35 µM	Leu517, Tyr396, Val382, Phe392, Thr430, Phe515

*reference molecule

### 3.3. Pharmacokinetics and drug-likeness properties

Table 4 shows the ADME properties of the polyphenols detected in the propolis samples. According to Lipinski, a compound under consideration should possess five properties in order to be selected as a potential drug - (a) molecular mass <500 Daltons, (b) high lipophilicity (expressed as LogP 5), (c) fewer than five hydrogen bond donors, (d) fewer than 10 hydrogen bond acceptors, and (e) molar refractivity between 40 and 130. The scanned eight flavonoid compounds used in this study were all found to satisfy Lipinski’s five conditions (Table 4). Other properties like pharmacokinetic, physicochemical and drug-likeness characteristics are shown in Table 4. The results indicated that all eight molecules have the potential to work effectively as novel drugs.

**Table 4:** ADME properties of ligands docked with SARS-CoV-2 Spike RBD and ACE-2 target proteins.

	(Lipinski’s Rule of Five)																	
Ligand name	Mol. weight	LogP	H-bond donor	H-bond acceptor	Molar Refractivity	Heavyatoms	Aromaticheavyatoms	Rotat.bonds	TPSA	ESOL Class	GIabsorption	BBBpermeant	Pgpsubstrate	BioAvail.Score	PAINSalerts	SyntheticAccessibility	Violations	DrugLikeliness
Pinocembrin	256.25	2.26	2	4	69.55	19	12	1	66.76 Å	soluble	High	Yes	No	0.55	0	2.96	No	Yes
Chrysin	254.24	2.5	2	4	71.97	19	16	1	70.67 Å	moderately soluble	High	Yes	No	0.55	0	2.93	No	Yes
CAPE	284.31	3.26	2	4	80.77	21	12	6	66.76 Å	moderately soluble	High	Yes	No	0.55	1	2.64	No	Yes
Hesperetin	302.28	1.91	3	6	78.06	22	12	2	66.76 Å	soluble	High	No	Yes	0.55	0	3.22	No	Yes
Ferulic acid	194.18	1.36	2	4	51.63	14	6	3	66.76 Å	soluble	High	Yes	No	0.85	0	1.93	No	Yes
t-Cinnamic acid	148.16	1.79	1	2	43.11	11	6	2	37.30 Å	soluble	High	Yes	No	0.85	0	1.67	No	Yes
p-coumaric acid	164.16	1.26	2	3	45.13	12	6	2	57.53 Å	soluble	High	Yes	No	0.85	0	1.61	No	Yes
Caffeic acid	180.16	0.93	3	4	47.16	13	6	2	77.76 Å	very soluble	High	Yes	No	0.56	1	1.81	No	Yes

Lipinski’s rule of five: Molecular weight (<500 Da), LogP (<5), H-bond donor (<5), H-bond acceptor (<10), molar refractivity (40-13).

### 3.4. In vitro inhibition studies

**Figure 10:**
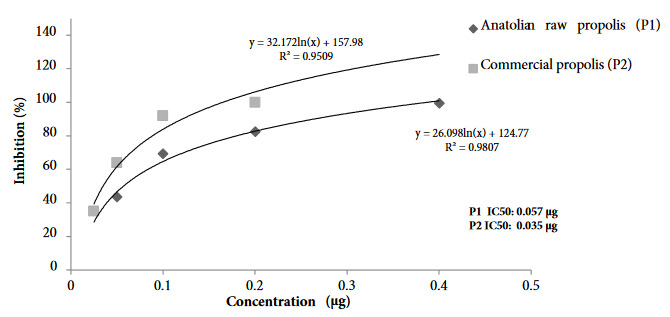
Inhibition curves (IC50) of P1 and P2 for SARS-CoV-2 Spike S1 protein/ACE-2 protein complex.

**Figure 11:**
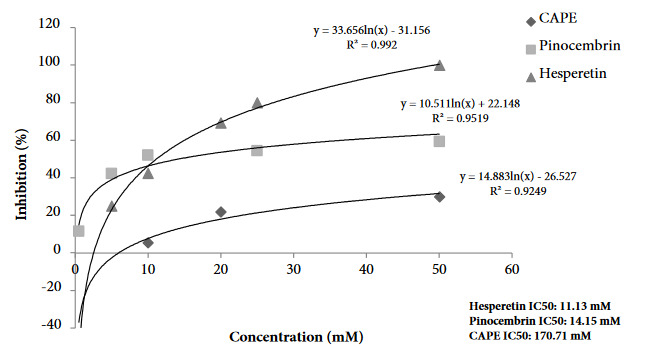
Inhibition curves (IC50) of CAPE, pinocembrin and hesperetin for SARS-CoV-2 Spike S1 protein/ACE-2 protein complex.

The binding of ACE-2 protein to SARS-CoV-2 spike S1 protein was studied for both EPEs using the inhibitor screening colorimetric assay kit (BPS Bioscience, 79954). The key to this ELISA assay is the high sensitivity of detection of ACE-2-Biotin protein by Streptavidin-HRP. This technique is based on the binding of the active ingredients of the propolis to this spike S1 protein/ACE-2 complex and inhibition of the binding of the enzyme-labeled second antibody to the protein. The presence of enzyme activity (horseradish peroxidase) indicates the absence of binding. The inhibition values are expressed in terms of the IC_50_ value and as the amount of the propolis that provides 50% inhibition. The relevant data are shown in Figures 10–11. The ability of specific flavonoids to inhibit the interaction of SARS-CoV-2 S1 spike protein and ACE-2 was also tested together with the propolis samples. The two EPE samples were found to cause inhibition of interaction of SARS-CoV-2 S1 spike protein: ACE-2 receptors, the degree of inhibition (IC_50_) varying depending on the propolis concentration. The IC_50_ value of the P2 sample was higher than that in the P1 sample.

The inhibition effects of five different concentrations of hesperetin, CAPE and pinocembrin were tested with the ELISA plate assay. The inhibition values varied depending on the concentration (Figure 10). Hesperetin emerged as the best inhibitor against the SARS-CoV-2 S1 spike protein and ACE-2, and had the lowest IC_50_value (11.13 mM), followed by pinocembrin and CAPE. When comparing in silico and in vitro study results, pinocembrin had a high inhibitory effect in the in silico study, whereas hesperetin was more active in the in vitro study.

## 4. Discussion

Propolis is a natural bee product and a very good source of polyphenols. The ideal extraction solvent for propolis rich in phenolic acid and flavonoids is 70% ethanol. The most characteristic analysis parameters for propolis are total polyphenol, total flavonoid and phenolic content analysis. The phenolic contents of the two ethanolic propolis samples in the present study were similar in terms of composition but differed in terms of quantities. The main reason for this difference is the amount of the raw propolis used initially when extracting with solvent (70%). In the P1 sample, 3 g of raw propolis was prepared at a ratio of 1:10 in 30 mL 70% alcohol. However, since the P2 sample was a commercial product, it was unclear how much raw propolis had been used, and it can only be stated with certainty that higher quantities were employed than the P1 sample. It was not known how much propolis was used in the commercial propolis sample, and the solid content was calculated to determine the solute content in the extract. As a result, it was determined that the amount of soluble matter was higher in the commercial sample, which indicates that cruder propolis was used in the commercial sample. However, the quality of raw propolis used in extraction is also an important parameter (Yeo et al., 2015). Reported TPC in Anatolian raw propolis samples varied from 115 mg GAE/g to 210 mg GAE/g in one study (Aliyazıcıoglu et al., 2011). It was also reported that TPC varied from 55.75 to 91.32 mg GAE/g in Brazilian propolis (Andrade et al., 2017), while in another study, TPC varied from 10 to 80 mg GAE/g in Azerbaijan propolis (Zehra et al., 2015). These results show that TPC is a critical quality criterion, and this quality also depends on the flora involved. 

The composition of propolis extracts depends on many factors such as the flora of the area where the raw propolis is collected, the time of collection and the extraction techniques. For this reason, it is not easy to standardize propolis extracts. As a matter of fact, it has been reported that the total amount of polyphenol found in Red propolis collected from different regions is between 150 and 220 mg GAE/g (Reis et al., 2020). Another study shows that the type and concentration of the solvent used in the preparation of propolis extracts affect the amount of TPC in the extract (Devequi-Nunes et al., 2018). As a matter of fact, in another experimental animal study conducted two years ago with the same commercial propolis sample used in the present study, it was reported that the propolis extract contained 102 mg GAE/mL (El Adaouia Taleb et al., 2020). 

The samples were also identified as acidic, (pH < 6.0), and the acidity was derived from the organic acids contained in the propolis. Caffeic acid, p-coumaric acid, and ferulic acids were detected in both samples. However, other studies have described gallic acid, caffeic acid, coumaric acid, ferulic acid and syringic acid, and protocatechuic acid as the major phenolic acids in propolis samples (Aliyazıcıoglu et al., 2011; Yeo et al., 2015). No gallic acid, protocatechuic acid, p-OH benzoic acid, or syringic acid were detected in any sample although this does not necessarily mean that there will be no gallic acid in Anatolian propolis (Keskin et al., 2019). Since these phenolic acids are highly polar compounds, they may not have switched to ethanol with a lower polarity than water. CAPE, pinocembrin, and chrysin were identified the most abundant flavonoids in both samples in the present study. CAPE is a polyphenol found mostly in propolis, with high quantities indicating a better quality of propolis, and possesses a wide range of bioactive properties such as antioxidant, anti-inflammatory, and anti-tumoral activities (Aliyazıcıoglu et al., 2011; Bankova et al., 2014; Venkateswara et al., 2017; Bankova et al., 2019). 

Pinocembrin, hesperetin, and chrysin were abundantly present flavonoids in the EPEs. Flavonoids are the most common and the largest plant polyphenolic obtained from the everyday plant-source diet (Chun et al., 2007). They have also been shown to be responsible for a variety of biological properties, such as antioxidant, antibacterial, antiviral, and anti-inflammatory activities. The estimated amount of flavonoids consumed in the daily diet is approximately 200 mg/day, consisting of 84% flavan-3-ols, flavanones (7.6%), flavonols (7%), anthocyanidins (1.6%), flavones (0.8%), and isoflavones (0.6%). However, epidemiological studies conducted in populations with flavonoid-rich diets have shown a lower incidence of cardiovascular damage (Cui et al., 2008). Studies have also described propolis as a very good source of flavonoids (Venkateswara et al., 2017; Kowacz and Pollack, 2020). The consumption of propolis as a food supplement thus provides high levels of polyphenols and flavonoids.

The antioxidant capacity of the EPEs was measured based on the FRAP test, a very simple and easy to apply method showing total antioxidant capacity. The higher the FRAP value measured based on the reduction ability of the Fe (III) TPTZ complex, the higher the antioxidant capacity (Can et al., 2015). In the present study, the antioxidant capacity of the commercial propolis sample (P2) was approximately close to the P1 sample. Although the P2 sample contains approximately twice as much TPC, it is thought that the close antioxidant capacity is due to the high antioxidant properties of some polyphenols in the phenolic profile of the P1 sample (Can et al. 2015; Kolaylı et al., 2020).

Molecular docking is a crucial tool for exploring the interactions between the target protein and a small molecule. Binding energy (kcal/mol) data make it possible to study and compare the binding affinities of different ligands/compounds with their corresponding target receptor molecules. Lower binding energy indicates a higher affinity of the ligand for the receptor. The ligand with the highest affinity can be selected as a potential drug for further investigation. For the present study, eight flavonoids with a broad range of biological activities, along with hydroxychloroquine, which exhibited efficacy against SARS-CoV-2, were selected as ligands in order to investigate their binding affinities with SARS-CoV-2 spike protein RBD and ACE-2 as target receptor proteins. All these eight polyphenols and one reference molecule were individually docked to the ACE-2 and SARS-CoV-2 spike RBD. Following successful docking of all the ligands used in these docking experiments, the results revealed significant interactions between the ligands and the target receptors. Four ligands (pinocembrin, chrysin, CAPE, and hesperetin) bound to the target protein ACE-2 more effectively than the reference molecule. Additionally, seven ligands (pinocembrin, chrysin, hesperetin, CAPE, ferulic acid, t-cinnamic acid, and caffeic acid) bound to the SARS-CoV-2 spike RBD more strongly than the reference molecule, hydroxychloroquine. The results of the docking study show that pinocembrin has the highest binding energy values, –8.58 kcal/mol for ACE-2 protein and –7.54 kcal/mol for SARS-CoV-2 Spike RBD, followed by chrysin with docking scores of –8.47 and –7.48 kcal/mol, respectively. Previous molecular docking studies involving propolis and Covid-19 reported that some propolis flavonoids exhibited high binding affinities to ACE-2 receptors and the virus spike protein. For example, quercetin and rutin have been reported to exhibit similar activity (Basu et al., 2020; Guler and Kara, 2020; Mady et al., 2020; Berretta et al., 2020). Another study compared the binding of 10 flavonoids in ethanolic extracts of propolis to ACE-II with that of MLN-4760, a known blocker of ACE-II (Guler et al., 2021). Rutin, CAPE, myricetin, quercetin, pinocembrin and hesperetin had stronger binding affinities to ACE-II than that of the reference molecule (Guler et al., 2021). In another study, docking analysis was performed on 22 propolis compounds against SARS-CoV-2 main protease (Mpro) and spike protein subunit 2 (S2), and four were found to exhibit strong binding affinities (Harisna et al., 2021).

Lipinski’s rule of five essentially determines the molecular properties of a compound, namely the primary requirement for being a potential drug, such as absorption, distribution, metabolism, and excretion (ADME). Generally, various parameters are used to evaluate potential interactions between a drug and other non-drug target molecules (Lipinski, 2004; Jayaram et al., 2012; Das, et al., 2020; Gupta et al., 2020). The suitability for a compound with a specific pharmacological or biological activity to be used as a potential drug is evaluated. The eight polyphenols detected in the propolis samples in the present study met the conditions specified by Lipinski, and other features were also compatible. We, therefore, suggest that the flavonoids have the potential to function effectively as novel drugs.

The aim of the present study, involving two propolis extracts, was to investigate the inhibition potential of ethanolic propolis extracts by binding to SARS-CoV-2 spike protein and ACE-2. In vitro study revealed that both samples caused inhibition, but that the P2 sample exhibited higher activity. We attributed this to the higher polyphenol content of the P2 sample. The finding that three polyphenols (hesperetin, pinocembrin and CAPE) studied separately resulted in inhibition of the virus shows that the active substances in propolis mostly derive from flavonoids. Since no previous in vitro studies have been published, we were unable to compare and discuss our results. None of the eight phenolic standards were tested in vitro by the ELISA KIT assay method, since the plate was limited to 96 well plates. Following in silico study, the in vitro inhibitions of the three flavonoids with the highest binding potential were examined. 

When comparing in silico study results with in vitro findings, pinocembrin, hesperetin, and CAPE were found to exhibit high binding affinities to the virus spike S1 protein and ACE-2 receptor. The in silico and in vitro studies, thus, support one another. In addition, the phenolic standards used in terms of ADME properties were found to exhibit high drug properties, and the results proved that propolis has a high potential in combating the Covid-19.

## 5. Conclusion

In this study, ethanolic Anatolia propolis extracts were observed, for the first time in the literature, to inhibit the Covid-19 virus in terms of binding spike S1 protein and ACE-2 receptor in both in vitro and in silico studies. However, there may be a need for more studies in the future.
